# Prognostic Factors in Acute Ischemic Stroke With a Decreased Estimated Glomerular Filtration Rate

**DOI:** 10.1002/brb3.71589

**Published:** 2026-07-09

**Authors:** Chang Hyeon Kim, Hyun Uk Lee, Byoung‐Soo Shin, Hyun Goo Kang

**Affiliations:** ^1^ Medical School Jeonbuk National University Jeonju South Korea; ^2^ Department of Neurology and Research Institute of Clinical Medicine Jeonbuk National University Jeonju South Korea; ^3^ Biomedical Research Institute Jeonbuk National University Medical School and Hospital Jeonju South Korea

**Keywords:** glomerular filtration rate, ischemic stroke, thrombectomy

## Abstract

**Introduction:**

Intra‐arterial thrombectomy (IAT), which is widely used to treat acute ischemic stroke, occasionally results in adverse outcomes. IAT‐associated risks in patients with decreased estimated glomerular filtration rate (eGFR) remain undefined. Therefore, this study aimed to investigate the effect of IAT on outcomes in patients with acute ischemic stroke and decreased eGFR and to explore other prognosis‐associated factors.

**Methods:**

This study included 309 patients with acute ischemic stroke and decreased eGFR to compare demographics, risk factors, and clinical outcomes based on IAT status. Factors affecting outcomes were investigated, and multivariate logistic regression analysis was performed to identify important determinants.

**Results:**

In a cohort of 309 patients with acute ischemic stroke and decreased eGFR, the factors associated with good outcomes, defined as a 3‐month modified Rankin Scale score of 0–2, included younger age, absence of IAT, and a higher eGFR. White matter hyperintensity, early neurological deterioration, and C‐reactive protein levels, which were significant in the univariate analysis, were not significant in the multivariate model.

**Conclusion:**

In patients with acute ischemic stroke and decreased eGFR, IAT receipt was associated with poorer 3‐month functional outcomes compared with no IAT. Older age and lower eGFR were independently associated with poorer prognosis, possibly reflecting increased vascular vulnerability and diminished neurorecovery capacity. Although IAT remains an important treatment option, these findings highlight the need for further research specific to AIS patients with decreased eGFR to support individualized clinical decision‐making.

## Graphical abstract

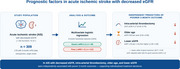

**Abbreviations**
ACC: American College of Cardiology
AHA: American Heart Association
AIS: acute ischemic stroke
CE: cardioembolism
CI: confidence interval
eGFR: estimated glomerular filtration rate
END: early neurological deterioration
HDL‐C: high‐density lipoprotein cholesterol
IAT: intra‐arterial thrombectomy
IVT: intravenous thrombolysis
KDIGO: Kidney Disease Improving Global Outcomes
LAA: large‐artery atherosclerosis
LDL‐C: low‐density lipoprotein cholesterol
mRS: modified Rankin Scale
NIHSS: National Institutes of Health Stroke Scale
SVO: small‐vessel occlusion
TICI: Thrombolysis in Cerebral Infarction
TOAST: Trial of Org 10172 in Acute Stroke Treatment
WMH: white matter hyperintensity

## 1 Introduction

Intra‐arterial thrombectomy (IAT) is a crucial treatment for acute ischemic stroke (AIS). IAT has significantly improved the long‐term outcomes of many patients with AIS and played a role in reducing mortality rates (McCarthy et al. 2019). However, approximately half of patients with AIS still experience poor long‐term outcomes after IAT (Olivot et al. 2022). Therefore, further research on IAT is needed to ensure appropriate treatment of patients.

Discussions regarding the implementation of IAT in patients with AIS and decreased estimated glomerular filtration rate (eGFR) are ongoing. Because contrast agents used during IAT exert toxic tubular effects and can induce renal ischemia, patients with decreased eGFR face a significantly elevated risk of contrast‐associated acute kidney injury, potentially leading to poor clinical outcomes (Mamoulakis et al. 2017; Shams and Mayrovitz 2021). In line with this, clinical data suggest that AIS patients with decreased eGFR tend to experience poor post‐IAT outcomes compared to those with normal renal function (Pan et al. 2020). However, these studies were limited because they only included patients who underwent IAT, hindering comparison between the outcomes of patients treated with IAT and those who were not. Additionally, studies comparing the outcomes of patients with AIS and renal dysfunction based on IAT status are limited and have often focused on patients undergoing dialysis (Saeed et al. 2014).

A baseline reduction in eGFR has been established as a potent independent risk factor that increases AIS incidence by up to twofold compared to individuals with normal renal function (Lee et al. 2010). Consequently, while a substantial proportion of patients with AIS present with decreased eGFR, clinical evidence evaluating how this renal impairment uniquely influences clinical outcomes depending on IAT status remains insufficient. Therefore, investigating the distinct clinical trajectories of AIS patients stratified by both renal function and IAT status is crucial to bridge this knowledge gap and optimize targeted therapeutic strategies.

## 2 Materials and Methods

### 2.1 Participant Selection

**FIGURE 1:**
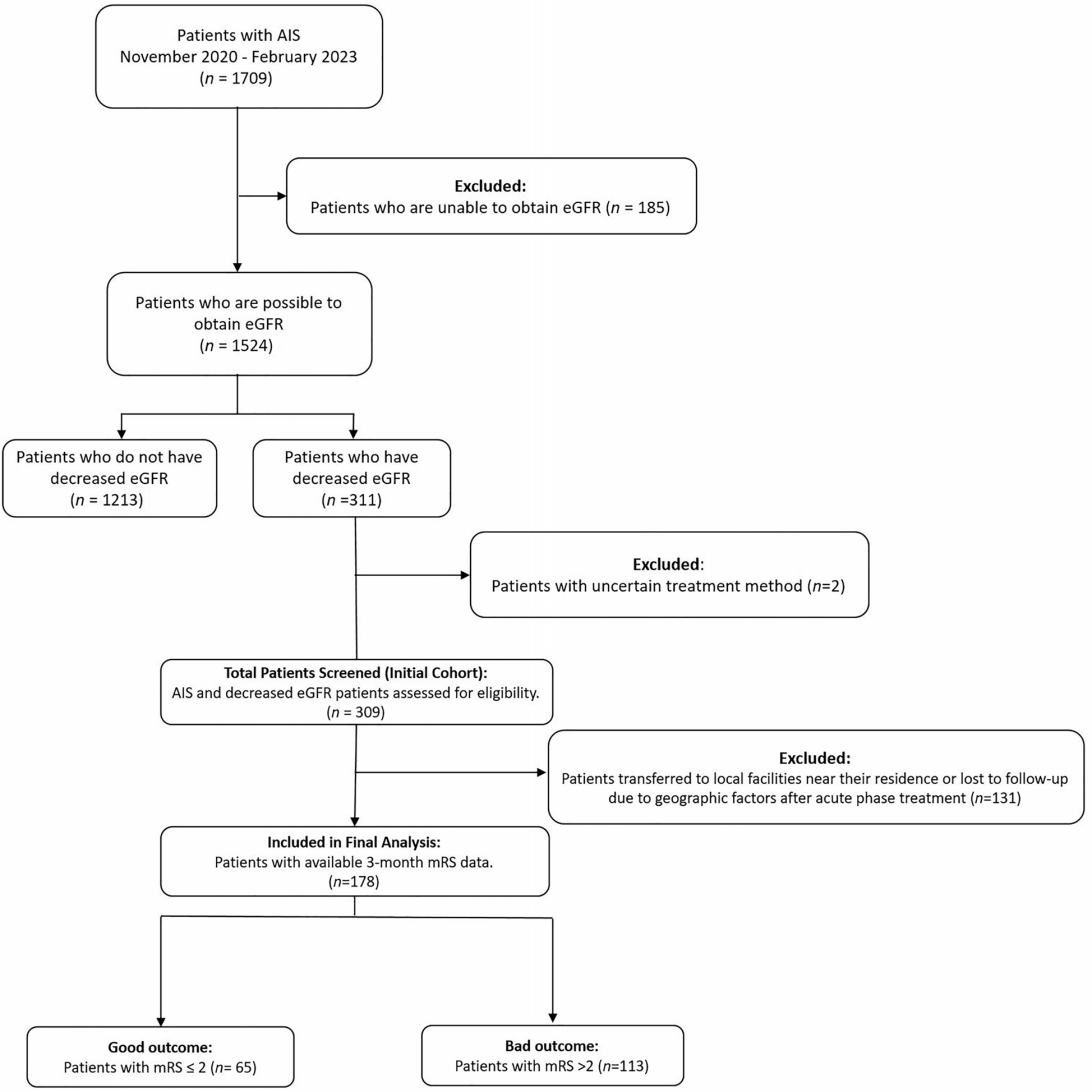
Study flowchart: (a) decreased eGFR population. AIS, acute ischemic stroke; eGFR, estimated glomerular filtration rate; IAT, intra‐arterial thrombectomy; mRS, modified Rankin scale.

This retrospective study included 1709 patients diagnosed with AIS and admitted to a regional tertiary hospital between 2020 and 2023. After excluding 185 patients without eGFR data, a total of 1524 patients were included in the final analysis, of whom 311 were identified as having decreased eGFR. After further excluding two patients with undetermined treatment status, a total of 309 patients with decreased eGFR were initially enrolled to compare baseline characteristics and clinical features. Among these, 178 patients (57.6%) who completed the 3‐month follow‐up with available modified Rankin Scale (mRS) scores were included in the outcome analysis, while the remaining 131 patients were excluded due to missing mRS data. To address potential selection bias, we also compared the baseline characteristics between the follow‐up cohort (*n* = 178) and those lost to follow‐up (*n* = 131); these results are provided in Table S1. In the follow‐up cohort, patients were further classified into the good outcome group (mRS ≤ 2, *n* = 65) and the poor outcome group (mRS > 2, *n* = 113) (Figure 1). This study was conducted in accordance with the Declaration of Helsinki and was approved by the institutional review board.

### 2.2 Data Assessment and Classifications

In the present study, decreased eGFR was defined according to the Kidney Disease Improving Global Outcomes (KDIGO) 2012 guidelines as an eGFR ˂ 60 mL/min/1.73 m^2^ at the time of admission (Stevens and Levin 2013). eGFR was estimated using serum creatinine levels, and the Chronic Kidney Disease Epidemiology Collaboration creatinine equation was used for this calculation (Inker et al. 2021). White matter hyperintensity (WMH) was assessed using the Fazekas scale, with periventricular white matter classified as Grade 0 if absent, Grade 1 if “caps” or pencil‐thin lining were present, Grade 2 if a smooth “halo” was observed, and Grade 3 if irregular periventricular signals extended to the deep white matter. Grade 0 indicated absence, Grade 1 indicated punctate foci, Grade 2 indicated the start of confluence, and Grade 3 indicated large confluent areas (Fazekas et al. 1987). Microbleeds were evaluated as follows: Grade 0, absent; Grade 1, 1–2 lesions; Grade 2, 3–10 lesions; and Grade 3, > 10 lesions (Gregoire et al. 2009). Thrombolysis was categorized into four groups: no thrombolysis, intravenous thrombolysis (IVT) only, both IVT and IAT, and IAT only. The Thrombolysis in Cerebral Infarction (TICI) scale was used to assess reperfusion, with Grade 0 indicating no perfusion, Grade 1 indicating minimal perfusion, Grade 2a indicating less than two‐thirds perfusion of the entire vascular territory, Grade 2b indicating full perfusion of the entire vascular territory but at a rate slower than the normal rate, and Grade 3 indicating complete perfusion (Fugate et al. 2013). Stroke subtypes were classified according to the Trial of Org 10172 in Acute Stroke Treatment (TOAST) criteria for large‐artery atherosclerosis (LAA), cardioembolism (CE), and small‐vessel occlusion (SVO) (Chen et al. 2012). Early neurological deterioration (END) was defined as the occurrence of new or worsening neurological symptoms within 3 weeks of stroke onset. The data collected included the National Institutes of Health Stroke Scale (NIHSS) score at admission and the mRS score 3 months after discharge.

### 2.3 Definition of Stroke Risk Factors

This study evaluated stroke risk factors such as hypertension, diabetes mellitus, dyslipidemia, and other related conditions. Hypertension was defined as a previous diagnosis of hypertension, use of antihypertensive medications, or measured systolic blood pressure ≥ 140 mmHg or diastolic blood pressure ≥ 90 mmHg in a stable state (Flack and Adekola 2020). Diabetes mellitus was defined as a previous diagnosis of diabetes with ongoing use of hypoglycemic agents or insulin, fasting blood glucose level ≥ 126 mg/dL, or postprandial blood glucose level ≥ 200 mg/dL at the time of admission (Blonde et al. 2022). Atrial fibrillation was defined as a paroxysmal supraventricular arrhythmia characterized by uncoordinated atrial activity and ineffective atrial contractions (Joglar et al. 2024). Dyslipidemia was defined as a previous diagnosis of dyslipidemia with ongoing statin therapy or meeting the following criteria according to the 2019 American College of Cardiology/American Heart Association (ACC/AHA) guidelines for the primary prevention of cardiovascular disease at the time of admission: (1) low‐density lipoprotein cholesterol (LDL‐C)  ≥ 130 mg/dL; (2) high‐density lipoprotein cholesterol (HDL‐C) < 40 mg/dL for men and < 50 mg/dL for women; or (3) triglycerides ≥ 175 mg/dL (Arnett et al. 2019). Coronary artery disease was confirmed based on a previous diagnosis of coronary artery disease, myocardial infarction, or suspected symptoms based on electrocardiography, exercise stress tests, or echocardiography. In addition, smoking status and alcohol consumption were assessed at the time of admission. Congestive heart failure was confirmed by a previous diagnosis or by suspected findings based on brain natriuretic peptide levels and echocardiography. Stroke history was defined as the occurrence of either ischemic or hemorrhagic stroke prior to admission.

### 2.4 Statistical Analysis

The primary outcome was defined as a poor outcome at 3 months, operationalized as an mRS score > 2 (including mRS score of 6 for patients who died). Patients with an mRS ≤ 2 at 3 months were classified as having a good outcome. Furthermore, factors affecting good outcomes in patients with decreased eGFR were examined. Descriptive statistics for the measured variables are presented as frequencies (percentages) for categorical variables and means ± standard deviations for continuous variables. The results of the multivariate logistic regression analyses are reported as *p*‐values, adjusted odds ratios (aORs), and 95% confidence intervals (CI). Categorical variables were analyzed using Pearson's chi‐squared or Fisher's exact test, whereas continuous variables were analyzed using Student's *t*‐test. Logistic regression analyses were performed to identify factors affecting good outcomes in patients with decreased eGFR. Univariate logistic regression analysis was performed to identify independent variables with *p* ≤ 0.05, followed by multivariate logistic regression analysis. Statistical significance was set at *p* < 0.05. All analyses were performed using SPSS (version 29.0; IBM Corp., Armonk, NY, USA).

## 3 Results

### 3.1 Differences in Patients With AIS and Decreased eGFR Based on the Presence or Absence of IAT

Of 309 patients with decreased eGFR, 52 (16.8%) underwent IAT. Compared with patients without IAT (*n* = 257), patients with IAT were older (81.29 ± 7.31 vs. 77.59 ± 9.49 years, *p* = 0.008) and had a higher prevalence of anterior lesions (80.8% vs. 62.8%, *p* = 0.013) but lower rates of posterior (19.2% vs. 37.2%) and brainstem lesions (1.9% vs. 14.2%, *p* = 0.014). Furthermore, patients with IAT exhibited more severe WMH (Fazekas scale score ≥ 3: 65.2% vs. 41.0%, *p* = 0.010). According to the TOAST classification, LAA was more common in patients with IAT (50.0% vs. 29.4%, *p* < 0.001), whereas CE and SVO were more prevalent in those without IAT (CE: 7.7% vs. 12.9%; SVO: 0.0% vs. 19.4%, *p* < 0.001). The rate of END was higher in patients without IAT; however, the NIHSS score at admission and the mRS score at 3 months were higher in patients with IAT (*p* < 0.001). Atrial fibrillation was significantly more common in patients who underwent IAT (51.9% vs. 19.8%, *p* < 0.001), whereas smoking was more prevalent in patients who did not undergo IAT (14.4% vs. 3.8%, *p* = 0.037). In addition, patients with IAT had significantly lower triglyceride levels than those without IAT (117.43 ± 57.99 vs. 152.34 ± 117.50 mg/dL, *p* = 0.003) (Table 1).

**TABLE 1:** Differences in patients with AIS and decreased eGFR based on the presence or absence of IAT.

	Patients without IAT (*n* = 257)	Patients with IAT (*n* = 52)	*p*‐value
Female sex	112 (43.6)	28 (53.8)	0.175
Lesion location			
Anterior	155 (62.8)	42 (80.8)	0.013
Posterior	92 (37.2)	10 (19.2)	
Brainstem lesion	35 (14.2)	1 (1.9)	0.014
Age (years)	77.59 ± 9.49 (257)	81.29 ± 7.31 (52)	0.008
White matter hyperintensity (Fazekas scale)			
0	20 (8.0)	1 (2.2)	0.010
1	41 (16.5)	2 (4.3)	
2	86 (34.5)	13 (28.3)	
3	102 (41.0)	30 (65.2)	
Microbleeds			
0	95 (41.3)	18 (38.3)	0.890
1	71 (30.9)	14 (29.8)	
2	38 (16.5)	10 (21.3)	
3	26 (11.3)	5 (10.6)	
TOAST			
LAA	73 (29.4)	26 (50.0)	< 0.001
CE	32 (12.9)	4 (7.7)	
SVO	48 (19.4)	0 (0.0)	
END	15 (6.3)	11 (23.9)	< 0.001
NIHSS score at admission	3 [1–5] (257)	11 [8–15] (52)	< 0.001
mRS score at 3 months	3 [2–4] (147)	4 [3–6] (31)	< 0.001
BMI	23.61 ± 3.50 (257)	24.02 ± 4.26 (51)	0.525
BMD	−1.99 ± 1.35 (30)	−1.86 ± 1.83 (7)	0.832
Onset time to arrival (s)	3481.18 ± 18,535.80 (200)	642.44 ± 1712.24 (25)	0.446
Last normal time to arrival (s)	1901.75 ± 6232.83 (51)	585.31 ± 628.82 (26)	0.288
**Stroke risk factors**			
Hypertension	198 (77.0)	44 (84.6)	0.227
Diabetes mellitus	132 (51.4)	20 (38.5)	0.090
Atrial fibrillation	51 (19.8)	27 (51.9)	< 0.001
Dyslipidemia	43 (16.7)	7 (13.5)	0.559
Previous stroke	69 (26.8)	19 (36.5)	0.158
Ischemic heart disease	31 (12.1)	10 (19.2)	0.168
Congestive heart failure	12 (4.7)	5 (9.6)	0.178
Smoking	37 (14.4)	2 (3.8)	0.037
Alcohol	30 (11.7)	5 (9.6)	0.669
**Laboratory findings**			
WBC (10^3^/µL)	8.48 ± 6.98 (257)	9.72 ± 3.17 (52)	0.214
Hb (g/dL)	11.66 ± 2.16 (257)	11.48 ± 2.28 (52)	0.581
Platelet ($10^{3}$/µL)	207.48 ± 78.41 (257)	195.29 ± 85.06 (52)	0.314
Na (mEq/L)	138.58 ± 3.46 (257)	140.13 ± 7.79 (52)	0.164
K (mEq/L)	4.38 ± 0.65 (257)	4.41 ± 1.73 (52)	0.914
CRP (mg/dL)	19.11 ± 37.16 (257)	50.61 ± 66.18 (52)	0.002
ESR (mm/h)	27.53 ± 24.52 (257)	26.31 ± 20.78 (51)	0.740
Calcium (mg/dL)	9.15 ± 0.81 (108)	8.79 ± 0.67 (30)	0.029
AST (U/L)	33.69 ± 61.14 (257)	49.72 ± 97.56 (52)	0.125
ALT (U/L)	22.57 ± 51.94 (257)	34.87 ± 87.67 (52)	0.332
ALP (U/L)	91.37 ± 54.53 (104)	78.18 ± 25.31 (33)	0.182
Albumin (g/dL)	3.99 ± 0.52 (257)	3.69 ± 0.52 (52)	< 0.001
BUN (mg/dL)	28.97 ± 14.85 (257)	32.85 ± 16.79 (52)	0.094
Creatinine (mg/dL)	1.96 ± 1.74 (257)	1.64 ± 0.92 (52)	0.056
GFR (mL/min)	40.45 ± 15.48 (257)	41.62 ± 15.25 (52)	0.620
Triglyceride (mg/dL)	152.34 ± 117.50 (229)	117.43 ± 57.99 (47)	0.003
HDL (mg/dL)	40.60 ± 11.59 (231)	42.60 ± 13.53 (47)	0.296
LDL (mg/dL)	92.77 ± 36.67 (229)	89.19 ± 35.96 (47)	0.542
Total cholesterol (mg/dL)	156.21 ± 45.33 (231)	147.95 ± 40.13 (47)	0.247
HbA1c (%)	6.37 ± 1.24 (246)	6.54 ± 2.08 (47)	0.462
TSH (uIU/mL)	2.59 ± 2.10 (108)	2.70 ± 1.93 (17)	0.838
fT4 (ng/dL)	15.96 ± 3.31 (106)	15.96 ± 2.99 (17)	0.998
Fibrinogen	350.24 ± 94.63 (245)	366.16 ± 88.55 (51)	0.270
Homocysteine	20.67 ± 31.39 (209)	11.47 ± 5.75 (39)	0.070
Uric acid (mg/dL)	5.68 ± 2.31 (98)	5.05 ± 1.51 (30)	0.085

Abbreviations: ALP, alkaline phosphatase; ALT, alanine aminotransferase; AST, aspartate aminotransferase; BMD, bone mineral density; BMI, body mass index; BUN, blood urea nitrogen; CE, cardioembolism; CRP, C‐reactive protein; END, early neurological deterioration; ESR, erythrocyte sedimentation rate; fT4, free thyroxine 4; GFR, glomerular filtration rate; Hb, hemoglobin; HbA1c, hemoglobin A1c; HDL, high‐density lipoprotein; IAT, intra‐arterial thrombectomy; IVT, intravenous thrombolysis; LAA, large‐artery atherosclerosis; LDL, low‐density lipoprotein; MRA, magnetic resonance angiography; mRS, modified Rankin scale; NIHSS, National Institutes of Health Stroke Scale; SUE, stroke of undetermined etiology; SVO, small‐vessel occlusion; TICI, Thrombolysis in Cerebral Infarction; TOAST, Trial of Org 10172 in Acute Stroke Treatment; tPA, tissue plasminogen activator; TSH, thyroid stimulating hormone; WBC, white blood cell.

### 3.2 Poor and Good Outcomes in Patients With AIS and Decreased eGFR

Among 178 patients with available 3‐month mRS data, 65 (36.5%) had good outcomes (mRS score ≤ 2) at 3 months, whereas 113 (63.5%) experienced poor outcomes (mRS score > 2). Patients with poor outcomes were older (78.48 ± 9.33 vs. 74.72 ± 8.16 years, *p* = 0.008) and had a significantly higher rate of IAT procedures (24.8% vs. 3%, *p* = 0.002). Furthermore, patients with decreased eGFR exhibited more severe WMH (Fazekas scale 3: 50.9% vs. 29.7%, *p* = 0.047) and higher incidences of CE and SVO, based on the TOAST classification. The poor outcome group demonstrated significantly more prevalent END (18.7% vs. 1.6%, *p* = 0.001) and higher NIHSS scores at admission (5 [3–10] vs. 2 [1–4], *p* < 0.001). Among the stroke risk factors, previous stroke was more common in the poor outcome group (37.2% vs. 21.5%, *p* = 0.031). In addition, the poor outcome group had higher CRP levels (34.12 ± 58.77 vs. 8.28 ± 20.31 mg/dL, *p* < 0.001), lower hemoglobin levels (11.57 ± 2.42 vs. 12.49 ± 1.93 g/dL, *p* = 0.006), lower albumin levels (3.82 ± 0.56 vs. 4.19 ± 0.53 g/dL, *p* < 0.001), and lower triglyceride levels (128.59 ± 61.93 vs. 185.95 ± 128.17 mg/dL, *p* = 0.002) than the good outcome group (Table 2).

**TABLE 2:** Poor and good outcomes in patients with AIS and decreased eGFR.

	Poor outcomes (*n* = 113)	Good outcomes (*n* = 65)	*p*‐value
Female sex	51 (45.1)	20 (30.8)	0.060
Lesion location			
Anterior	76 (67.3)	38 (60.3)	0.356
Posterior	37 (32.7)	25 (39.7)	
Brainstem lesion	16 (14.2)	8 (12.7)	0.787
Age (years)	78.48 ± 9.33 (113)	74.72 ± 8.16 (65)	0.008
White matter hyperintensity (Fazekas scale)			
0	5 (4.6)	6 (9.4)	0.047
1	13 (12.0)	12 (18.8)	
2	35 (32.4)	27 (42.2)	
3	55 (50.9)	19 (29.7)	
Microbleeds			
0	40 (39.2)	25 (42.4)	0.372
1	29 (28.4)	21 (35.6)	
2	21 (20.6)	6 (10.2)	
3	12 (11.8)	7 (11.9)	
Thrombolysis			
None	76 (67.3)	56 (86.2)	0.002
IVT	9 (8.0)	7 (10.8)	
IVT + IAT	7 (6.2)	1 (1.5)	
IAT	21 (18.6)	1 (1.5)	
Post‐TICI			
0	2 (10.5)	0 (0.0)	0.657
1	2 (10.5)	0 (0.0)	
2A	3 (15.8)	0 (0.0)	
2B	4 (21.1)	0 (0.0)	
3	8 (42.1)	2 (100.0)	
TOAST			
LAA	38 (33.6)	22 (34.9)	0.015
CE	8 (7.1)	8 (12.7)	
SVO	13 (11.5)	16 (25.4)	
END	20 (18.7)	1 (1.6)	0.001
NIHSS at admission	5 [3–10] (113)	2 [1–4] (64)	< 0.001
mRS at 3 months	4 [3–5] (113)	1 [1–2] (65)	< 0.001
BMI	23.77 ± 3.78 (113)	24.49 ± 3.14 (64)	0.202
BMD	−2.31 ± 1.13 (13)	−1.26 ± 1.13 (5)	0.098
Onset time to arrival (s)	2415.92 ± 4035.70 (76)	6258.53 ± 33,765.48 (59)	0.327
Last normal time to arrival (s)	2202.74 ± 7659.09 (34)	1034.75 ± 510.86 (4)	0.765
Onset‐to‐tPA time	491.75 ± 899.28 (8)	160.67 ± 56.16 (9)	0.333
Onset‐to‐puncture time	3621.17 ± 9648.17 (18)	227.50 ± 24.75 (2)	0.633
Onset‐to‐reperfusion time	3828.56 ± 9649.13 (18)	233.50 ± 27.58 (2)	0.633
**Stroke risk factors**			
Hypertension	87 (77.0)	48 (73.8)	0.637
Diabetes mellitus	57 (50.4)	35 (53.8)	0.662
Atrial fibrillation	31 (27.4)	12 (18.5)	0.178
Dyslipidemia	13 (11.5)	14 (21.5)	0.072
Previous stroke	42 (37.2)	14 (21.5)	0.031
Ischemic heart disease	15 (13.4)	13 (20.0)	0.246
Congestive heart failure	7 (6.2)	1 (1.5)	0.149
Smoking	13 (11.5)	12 (18.5)	0.198
Alcohol	12 (10.6)	10 (15.4)	0.352
**Laboratory findings**			
WBC (10^3^/µL)	8.74 ± 4.62 (113)	7.52 ± 2.62 (65)	0.026
Hb (g/dL)	11.57 ± 2.42 (113)	12.49 ± 1.93 (65)	0.006
Platelet ($10^{3}$/µL)	198.51 ± 84.57 (113)	205.26 ± 63.05 (65)	0.546
Na (mEq/L)	139.12 ± 5.96 (113)	138.65 ± 2.72 (65)	0.475
K (mEq/L)	4.46 ± 1.32 (113)	4.38 ± 0.49 (65)	0.644
CRP (mg/dL)	34.12 ± 58.77 (113)	8.28 ± 20.31 (65)	< 0.001
ESR (mm/h)	29.06 ± 24.22 (112)	25.31 ± 25.21 (65)	0.329
Calcium (mg/dL)	8.93 ± 0.75 (58)	9.40 ± 0.94 (27)	0.016
AST (U/L)	47.94 ± 109.21 (113)	26.63 ± 10.23 (65)	0.042
ALT (U/L)	33.98 ± 96.70 (113)	20.88 ± 9.57 (65)	0.278
ALP (U/L)	87.43 ± 43.76 (61)	81.46 ± 31.09 (26)	0.531
Albumin (g/dL)	3.82 ± 0.56 (113)	4.19 ± 0.53 (65)	< 0.001
BUN (mg/dL)	30.73 ± 15.46 (113)	28.77 ± 14.18 (65)	0.401
Creatinine (mg/dL)	2.07 ± 1.76 (113)	1.79 ± 1.35 (65)	0.236
GFR (mL/min)	38.33 ± 16.63 (113)	42.47 ± 13.15 (65)	0.069
Triglyceride (mg/dL)	128.59 ± 61.93 (97)	185.95 ± 128.17 (61)	0.002
HDL (mg/dL)	40.29 ± 12.49 (97)	41.29 ± 11.96 (62)	0.617
LDL (mg/dL)	91.80 ± 36.90 (97)	93.73 ± 38.38 (60)	0.754
Total cholesterol (mg/dL)	151.30 ± 44.64 (98)	165.60 ± 48.30 (61)	0.059
HbA1c (%)	6.29 ± 1.60 (103)	6.64 ± 1.33 (63)	0.141
TSH (uIU/mL)	2.63 ± 2.33 (51)	2.37 ± 1.39 (25)	0.598
fT4 (ng/dL)	15.66 ± 3.47 (50)	15.31 ± 2.31 (25)	0.613
Fibrinogen	347.96 ± 90.39 (110)	349.54 ± 94.88 (61)	0.915
Homocysteine	17.24 ± 9.63 (88)	17.89 ± 6.43 (54)	0.660
Uric acid (mg/dL)	5.29 ± 2.48 (55)	5.84 ± 2.02 (27)	0.318

Abbreviations: ALP, alkaline phosphatase; ALT, alanine aminotransferase; AST, aspartate aminotransferase; BMD, bone mineral density; BMI, body mass index; BUN, blood urea nitrogen; CE, cardioembolism; CRP, C‐reactive protein; END, early neurological deterioration; ESR, erythrocyte sedimentation rate; GFR, glomerular filtration rate; Hb, hemoglobin; HbA1c, hemoglobin A1c; HDL, high‐density lipoprotein; IVT, intravenous thrombolysis; IAT, intra‐arterial thrombectomy; LAA, large‐artery atherosclerosis; LDL, low‐density lipoprotein; mRS, modified Rankin scale; NIHSS, National Institutes of Health Stroke Scale; SUE, stroke of undetermined etiology; SVO, small‐vessel occlusion; T4, thyroxine 4; TICI, Thrombolysis in Cerebral Infarction; TOAST, Trial of Org 10172 in Acute Stroke Treatment; tPA, tissue plasminogen activator; TSH, thyroid stimulating hormone; WBC, white blood cell.

### 3.3 Factors Affecting Good Outcomes in Patients With AIS and Decreased eGFR

In a cohort of 309 patients with decreased eGFR, logistic regression analysis was conducted to identify factors associated with good outcomes (mRS = ≤ 2) at 3 months. Univariate analysis revealed that younger age (OR, 0.955; 95% CI, 0.922–0.989; *p* = 0.010), less severe WMH (OR, 2.548; 95% CI, 1.276–4.735; *p* = 0.007), absence of IAT (OR, 0.092; 95% CI, 0.021–0.400; *p* = 0.001), absence of END (OR, 0.070; 95% CI, 0.009–0.537; *p* = 0.010), CRP levels (OR, 0.981; 95% CI, 0.968–0.994; *p* = 0.005), and a higher eGFR (OR, 1.018; 95% CI, 0.997–1.039; *p* = 0.089) were associated with good outcomes. Multivariate analysis confirmed that younger age (aOR, 0.951; 95% CI, 0.910–0.993; *p* = 0.024), absence of IAT (aOR, 0.170; 95% CI, 0.036–0.792; *p* = 0.024), and a higher eGFR (aOR, 1.031; 95% CI, 1.002–1.060; *p* = 0.034) remained significant predictors of good outcomes. Variables such as WMH (aOR, 1.883; 95% CI, 0.891–3.982; *p* = 0.097), END (aOR, 0.262; 95% CI, 0.029–2.357; *p* = 0.232), and CRP levels (aOR, 0.991; 95% CI, 0.977–1.006; *p* = 0.230), which were significant in the univariate analysis, were not statistically significant in the multivariate model (Table 3).

**TABLE 3:** Logistic regression analysis of factors affecting good outcomes in patients with AIS and decreased eGFR.

Variables	Univariate analysis		Multivariate analysis	
	Crude OR (95% CI)	*p*‐value	Adjusted OR (95% CI)	*p*‐value
Age (years)	0.955 (0.922–0.989)	0.010	0.951 (0.910–0.993)	0.024
White matter hyperintensity (Fazekas scale)	2.548 (1.276–4.735)	0.007	1.883 (0.891–3.982)	0.097
IAT	0.092 (0.021–0.400)	0.001	0.170 (0.036–0.792)	0.024
END	0.070 (0.009–0.537)	0.010	0.262 (0.029–2.357)	0.232
CRP (mg/dL)	0.981 (0.968–0.994)	0.005	0.991 (0.977–1.006)	0.230
GFR (mL/min)	1.018 (0.997–1.039)	0.089	1.031 (1.002–1.060)	0.034

*Note*: No. of events analyzed = 65 (good).

Abbreviations: CI, confidence interval; CRP, C‐reactive protein; END, early neurological deterioration; GFR, glomerular filtration rate; IAT, intra‐arterial thrombectomy; OR, odds ratio.

## 4 Discussion

IAT is a critical intervention for improving patient outcomes in AIS. However, clear evidence regarding the outcomes of IAT use in patients with ischemic stroke and decreased eGFR is lacking, particularly considering the use of contrast agents during the procedure. Previous studies have largely focused on patients with renal dysfunction who underwent IAT, especially those on dialysis, and direct comparisons between IAT‐treated and non‐IAT‐treated patients are limited (Saeed et al. 2014). Therefore, further research on IAT in patients with ischemic stroke and decreased eGFR is warranted. In the present study, we investigated outcomes in patients with ischemic stroke and decreased eGFR by comparing the IAT and non‐IAT groups. In addition, we identified factors that affect the prognosis of patients with ischemic stroke and decreased eGFR.

First, the prognosis of patients with ischemic stroke and decreased eGFR who underwent IAT was poor. This finding contrasts with the generally positive effect of IAT on outcomes in patients with ischemic stroke, for whom it is recognized as a crucial and beneficial treatment (Goyal et al. 2016; Jovin et al. 2015). In some circumstances, thrombectomy may cause arterial collapse or traction, which can make embolus removal more difficult, lead to vascular injury, or result in residual occlusion, thereby contributing to poorer neurological outcomes (Liu, Gebrezgiabhier, and Reddy, et al. 2022). In addition, arterial collapse is more likely in cases with poor collateral status (Liu, Gebrezgiabhier, and Reddy, 2022; Liu, Gebrezgiabhier, and Zheng, 2022b), and decreased eGFR has been linked to poor collateral status (Yao et al. 2021). Decreased eGFR is associated with risk factors such as uremic toxins and oxidative stress (Toyoda and Ninomiya 2014), all of which may promote vascular injury and endothelial dysfunction. Moreover, renal impairment is associated with decreased nitric oxide levels (Mogi and Horiuchi 2011). Nitric oxide plays a role in regulating microcirculation and the blood–brain barrier; consequently, a reduction in nitric oxide may contribute to microvascular dysfunction in the brain. Furthermore, a decreased eGFR has been associated with systemic hyperviscosity (Celik et al., 2017), suggesting a possible vulnerability to microvascular perfusion insufficiency. Notably, such hyperviscosity‐related rheological disturbances—including sluggish cerebral flow and in situ thrombosis of small vessels—have been recently reported in the context of ischemic processes (Wolie et al. 2026). Consequently, the potential interplay between a decreased eGFR and hyperviscosity‐mediated microvascular stagnation might be a factor worth considering in relation to suboptimal post‐IAT outcomes. These factors may contribute to the development of cerebral vessels with poor collateral status in patients with decreased eGFR, ultimately resulting in poor outcomes following IAT.

In this study, among patients presenting with a decreased baseline eGFR, lower eGFR was associated with poor outcomes following ischemic stroke. As previously discussed, renal dysfunction is associated with vascular injury, cardiovascular risk factors, and arterial calcification (Go et al. 2004). Furthermore, because the kidneys use the same vasoregulatory mechanisms as the brain (Ghoshal and Freedman 2019), reduced renal function may parallel subtle disruptions in cerebral vasoregulation. Such physiological alterations might be related to fluctuations in cerebral blood flow, which are frequently observed in the context of suboptimal clinical outcomes after AIS (Sedaghat et al. 2016). Furthermore, patients with ischemic stroke and a lower eGFR generally have poor outcomes, which is consistent with the findings of the present study (Nagaraja et al. 2023).

Old age was associated with poor outcomes in patients with ischemic stroke and decreased eGFR. Age‐related conditions such as hypertension, diabetes mellitus, and atrial fibrillation increase the risk of stroke and worsen the prognosis (Roy‐O'Reilly and McCullough 2018). Moreover, the aging process involves a series of changes, including decreased nitric oxide levels and increased endothelin levels, which affect arterial structure and function (Jani and Rajkumar 2006). When such age‐related vascular changes accompany AIS in patients with decreased eGFR, clinical outcomes may be particularly unfavorable following IAT. However, some previous studies have still recommended the use of IAT in older patients with AIS (Goyal et al. 2016; Jovin et al. 2015). Thus, the recommendations may not fully account for the compounding effect of renal impairment in specific subpopulations.

In this study, WMH, END, and CRP were significantly associated with outcomes in univariate analysis; however, these associations did not remain significant in the multivariate model. This attenuation may be explained by the fact that these variables largely reflect cumulative systemic vulnerability and stroke severity—factors that overlap considerably with age, IAT status, and eGFR. Additionally, the very small number of END events among patients with good outcomes likely resulted in wide CIs and reduced statistical power. Overall, among patients with AIS and decreased eGFR, advanced age, impaired renal function, and the need for IAT—reflecting more severe occlusions—are major determinants of 3‐month outcomes.

Although some previous studies have supported the use of IAT in patients with AIS and renal dysfunction when alternative treatments are unavailable (Kelly et al. 2021), evidence remains limited. Saeed et al. (2014) have reported that IAT‐treated patients with stroke on dialysis had lower mortality rates and less disability at discharge than non‐IAT‐treated patients with stroke on dialysis. While their findings highlight the immediate, short‐term survival benefit of IAT at the acute discharge phase, our results based on 3‐month mRS scores indicate a more comprehensive measure of longer‐term outcome (ElHabr et al. 2021). This discrepancy may be attributable to differences in follow‐up duration and the specific endpoints evaluated. IAT can provide immediate procedural success that may reduce acute‐phase mortality, as observed by Saeed et al. (2014). Over a longer period, a decreased baseline eGFR is consistently associated with oxidative stress, vascular injury, inflammation, and systemic hyperviscosity, which might collectively contribute to poor 3‐month clinical outcomes (Celik et al., 2017; Toyoda and Ninomiya 2014; Wolie et al. 2026).

Despite its findings, several limitations of this study warrant consideration. First, its retrospective, single‐center design introduces inherent selection bias and limits external validity. Second, baseline renal function was evaluated using a single admission eGFR measurement. While pragmatic for acute‐phase decisions and widely accepted in stroke research (Yahalom et al. 2009; Shimizu et al. 2011), this snapshot cannot definitively differentiate stable chronic impairment from acute kidney injury and may be transiently confounded by dehydration or systemic stress. Third, the high rate of patients lost to 3‐month follow‐up (42.4%) poses a potential risk for attrition bias. Although baseline characteristics were broadly similar between the followed and lost groups (Table S1), unmeasured systematic differences cannot be entirely ruled out. If patients lost to follow‐up disproportionately experienced distinct functional trajectories, our reported 3‐month poor outcome rate (63.5%) could be over‐ or underestimated, thereby limiting the generalizability and robustness of our final conclusions. Fourth, the multivariable model relied partly on univariate significance (*p* < 0.05) for variable selection. Nevertheless, with 65 good outcomes (limiting event class) among 178 patients, the six‐variable model yielded an event‐per‐variable (EPV) ratio of 10.83, satisfying the traditional threshold (EPV ≥ 10) to prevent overfitting. Crucially, however, our IAT cohort was significantly older and presented with greater baseline severity (higher NIHSS scores, higher atrial fibrillation prevalence, and severe WMH). This imbalance introduces a substantial risk of confounding by indication. Therefore, the observed link between IAT and poorer outcomes likely reflects the underlying severity of the index stroke rather than a direct harmful effect of the procedure. Consequently, these findings should be interpreted as hypothesis‐generating associations rather than causal evidence; future large‐scale, prospective, and multiethnic studies employing clinically guided modeling or propensity‐score‐based analysis are warranted to further validate the role of IAT in AIS patients with decreased eGFR.

In conclusion, our findings suggest that IAT is associated with poorer 3‐month outcomes in AIS patients with decreased eGFR compared to those who did not receive the treatment. Our analysis indicates that age, IAT status, and eGFR are significant factors linked to prognosis in this population. It is hypothesized that impaired renal function and advanced age may be associated with increased vascular vulnerability and diminished neurorecovery capacity, which could contribute to the observed associations. Notably, while most existing literature examines renal function within IAT‐treated cohorts, clinical data comparing IAT and non‐IAT groups specifically within the AIS‐decreased eGFR population remains limited. Our findings suggest a potential divergence from general stroke population trends, indicating that the association between IAT and clinical outcomes might be attenuated in this specific subpopulation. Given that current guidelines primarily reflect the general population, our results underscore the value of further research specific to AIS patients with decreased eGFR to support tailored clinical decision‐making.

## Author Contributions

**Chang Hyeon Kim**: conceptualization, formal analysis, investigation, writing – original draft. **Hyun Uk Lee**: conceptualization, formal analysis, investigation, writing – original draft. **Byoung‐Soo Shin**: formal analysis, investigation, writing – review and editing. **Hyun Goo Kang**: conceptualization, formal analysis, investigation, writing – review and editing, supervision.

## Funding

The authors have nothing to report.

## Ethics Statement

This study was approved by the Institutional Review Board of Jeonbuk National University Hospital (2024‐10‐018). The requirement for informed consent was waived owing to the retrospective nature of the study.

## Consent

The authors have nothing to report.

## Conflicts of Interest

The authors declare no conflicts of interests.

## Supporting information




**Supplementary Table**: brb371589‐sup‐0001‐TableS1.docx

## Data Availability

The datasets used and/or analyzed in the present study are available from the corresponding author upon reasonable request.
