# Supplementary material for: Prognostic Factors in Acute Ischemic Stroke With a Decreased Estimated Glomerular Filtration Rate
Source: Brain Behav. 2026 Jul 9;16(7):e71589. doi: 10.1002/brb3.71589 (PMC13347158; doi:10.1002/brb3.71589)
Supplement: Supplementary file 1 — Supplementary Table: brb371589‐sup‐0001‐TableS1.docx [file BRB3-16-e71589-s001.docx]

**Supplementary Table 1.** Baseline characteristics of patients with chronic kidney disease (CKD) according to the availability of 3-month modified Rankin Scale (mRS) data

|  | **Without 3-month mRS data (n = 133)** | **with 3-month mRS data (n = 178)** | ***P-value*** |
| --- | --- | --- | --- |
| Female sex | 71 (53.4) | 71 (39.9) | *0.018* |
| Lesion location |  |  | *0.770* |
| Anterior | 83 (66.4) | 114 (64.8) |  |
| Posterior | 42 (33.6) | 62 (35.2) |  |
| Brainstem lesion | 12 (9.6) | 24 (13.6) | *0.288* |
| Age (years) | 79.84 ± 9.31 | 77.11 ± 9.08 | *0.010* |
| White matter hyperintensity (Fazekas scale) |  |  | *0.667* |
| 0 | 10 (8.0) | 11(6.4) |  |
| 1 | 18 (14.4) | 25 (14.5) |  |
| 2 | 37 (29.6) | 62 (36.0) |  |
| 3 | 60 (48.0) | 74 (43.0) |  |
| Microbleeds |  |  | *0.921* |
| 0 | 48 (40.7) | 65 (40.4) |  |
| 1 | 35 (29.7) | 50 (31.1) |  |
| 2 | 23 (19.5) | 27 (16.8) |  |
| 3 | 12 (10.2) | 19 (11.8) |  |
| Thrombolysis |  |  | *0.439* |
| None | 102 (77.9) | 131 (73.6) |  |
| IVT | 8 (6.1) | 16 (9.0) |  |
| IVT + IAT | 9 (6.9) | 8 (4.5) |  |
| IAT | 12 (9.2) | 23 (12.9) |  |
| post TICI |  |  | *0.043* |
| 0 | 4(23.5) | 2 (9.5) |  |
| 1 | 0 (0.0) | 2 (9.5) |  |
| 2A | 0 (0.0) | 3 (14.3) |  |
| 2B | 9 (52.9) | 4 (19.0) |  |
| 3 | 4 (23.5) | 10 (47.6) |  |
| TOAST |  |  | *0.270* |
| LAA | 39 (31.0) | 60 (34.1) |  |
| CE | 21 (16.7) | 16 (9.1) |  |
| SVO | 19 (15.1) | 29 (16.5) |  |
| Other | 47 (37.3) | 71 (40.3) |  |
| END | 5 (4.4) | 21 (12.4) | *0.022* |
| NIHSS at admission | 5 [2-10] | 3 [2-6] | *0.058* |
| BMI | 23.15 ± 3.68 | 24.03 ± 3.57 | *0.033* |
| BMD | -1.91 ± 1.64 | -2.02 ± 1.20 | *0.824* |
| Onset time to arrival (seconds) | 722 [144-2880] | 872 [167-2880] | *0.334* |
| Last normal time to arrival (seconds) | 789 [500-1134] | 709 [280-1049] | *0.304* |
| Onset to tPA time | 148 [128-182] | 163 [117-212] | *0.366* |
| Onset to puncture time | 188 [151-306] | 319 [199-1126] | *0.297* |
| Onset to reperfusion time | 194 [153-308] | 324 [210-1133] | *0.297* |
| **Stroke risk factors** |  |  |  |
| Hypertension | 108 (81.2) | 135 (75.8) | *0.258* |
| Diabetes mellitus | 61 (45.9) | 92 (51.7) | *0.310* |
| Atrial fibrillation | 36 (27.1) | 43 (24.2) | *0.560* |
| Dyslipidemia | 23 (17.3) | 27 (15.2) | *0.614* |
| Previous stroke | 33 (24.8) | 56 (31.5) | *0.199* |
| Ischemic heart disease | 13 (9.8) | 28 (15.8) | *0.120* |
| Congestive heart failure | 9 (6.8) | 8 (4.5) | *0.383* |
| Smoking | 14 (10.5) | 25 (14.0) | *0.354* |
| Alcohol | 13 (9.8) | 22 (12.4) | *0.475* |
| **Laboratory findings** |  |  |  |
| WBC (10³/μL) | 9.21 ± 8.78 | 8.30 ± 4.04 | *0.223* |
| Hb (g/dL) | 11.26 ± 1.95 | 11.91 ± 2.29 | *0.009* |
| Platelet (/μL) | 212.07 ± 82.88 | 200.98 ± 77.29 | *0.226* |
| Na (mEq/L) | 138.72 ± 3.74 | 138.94 ± 5.02 | *0.668* |
| K (mEq/L) | 4.33 ± 0.60 | 4.43 ± 1.09 | *0.331* |
| CRP (mg/dL) | 23.72 ± 36.90 | 24.68 ± 49.91 | *0.845* |
| ESR (mm/h) | 26.91 ± 22.97 | 27.68 ± 24.59 | *0.778* |
| Calcium (mg/dL) | 9.06 ± 0.71 | 9.08 ± 0.84 | *0.929* |
| AST (U/L) | 31.10 ± 25.59 | 40.16 ± 87.70 | *0.249* |
| ALT (U/L) | 18.38 ± 12.42 | 29.20 ± 77.39 | *0.112* |
| ALP (U/L) | 93.84 ± 62.24 | 85.64 ± 40.30 | *0.349* |
| Albumin (g/dL) | 3.92 ± 0.47 | 3.96 ± 0.58 | *0.565* |
| BUN (mg/dL) | 29.14 ± 15.48 | 30.02 ± 15.00 | *0.613* |
| Creatinine (mg/dL) | 1.83 ± 1.63 | 1.96 ± 1.62 | *0.460* |
| GFR (mL/min) | 41.76 ± 15.13 | 39.84 ± 15.54 | *0.279* |
| Triglyceride (mg/dL) | 139.84 ± 126.00 | 150.73 ± 97.00 | *0.417* |
| HDL (mg/dL) | 41.13 ± 11.59 | 40.68 ± 12.26 | *0.754* |
| LDL (mg/dL) | 91.15 ± 35.78 | 92.54 ± 37.36 | *0.755* |
| Total cholesterol (mg/dL) | 151.53 ± 42.25 | 156.81 ± 46.46 | *0.330* |
| HbA1c (%) | 6.37 ± 1.26 | 6.42 ± 1.51 | *0.754* |
| TSH (uIU/mL) | 2.75 ± 2.20 | 6.42 ± 1.51 | *0592* |
| fT4 (ng/dL) | 16.62 ± 3.34 | 15.54 ± 3.12 | *0.068* |
| Fibrinogen | 358.95 ± 96.51 | 348.53 ± 91.74 | *0.343* |
| Homocysteine | 21.38 ± 43.22 | 17.49 ± 8.54 | *0.360* |
| Uric acid (mg/dL) | 5.60 ± 1.80 | 5.47 ± 2.34 | *0.753* |

IVT, intravenous thrombolysis; IAT, intra-arterial thrombectomy; TICI, Thrombolysis in Cerebral Infarction; TOAST, Trial of Org 10172 in Acute Stroke Treatment; LAA, large-artery atherosclerosis; CE, cardioembolism; SVO, small-vessel occlusion; SUE, stroke of undetermined etiology; END, early neurological deterioration; NIHSS, National Institutes of Health Stroke Scale; mRS, modified Rankin scale; BMI, body mass index; BMD, bone mineral density; tPA, tissue plasminogen activator; WBC, white blood cell; Hb, hemoglobin; CRP, C-reactive protein; ESR, erythrocyte sedimentation rate; BUN, blood urea nitrogen; GFR, glomerular filtration rate; AST, aspartate aminotransferase; ALT, alanine aminotransferase; ALP, alkaline phosphatase; HDL, high-density lipoprotein; LDL, low-density lipoprotein; HbA1c, hemoglobin A1c; TSH, thyroid stimulating hormone; T4, thyroxine 4
